# Traumatic false aneurysm of the distal peroneal artery with associated anatomic anomaly: a case report

**DOI:** 10.4076/1757-1626-2-8605

**Published:** 2009-07-20

**Authors:** Michael J Ramdass

**Affiliations:** Department of Surgery, General HospitalPort-of-Spain, TrinidadWest Indies

## Abstract

Football and ankle soft tissue injuries are common occurrences. However, traumatic peroneal false aneurysm is quite a rare entity with only a handful of cases reported in the literature. A case of traumatic false aneurysm of the distal peroneal artery is described in which an anatomic anomaly of the distal peroneal artery crossing the ankle joint may have been a predisposing factor. A technique for surgical approach is also described for the particular location of the lesion.

## Case presentation

**Figure 1.**
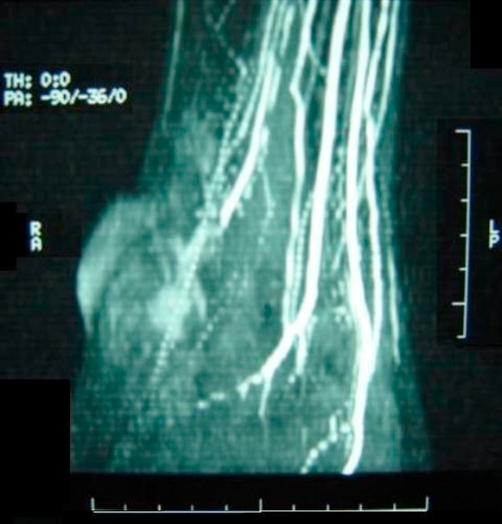
Anomalous peroneal artery, crossing the ankle joint and consisting of several large branches forming an anastomosis in the region with associated false aneurysm.

A 20-year-old Trinidadian gentleman of african-descent presented to orthopaedics after sustaining an injury to the right ankle whilst playing football. The injury involved inversion of the ankle joint with pain, swelling and tenderness in the region of the lateral malleolus. There was no notable neurovascular injury and radiographs were normal. The patient was advised rest and elevation and the joint immobilised for 2 weeks. At the first outpatient visit, it was noted that a pulsatile swelling had developed in the region of the lateral malleolus and the skin over the swelling was partially necrotic. A diagnosis of a false aneurysm was made and confirmed on duplex scanning. A magnetic resonance angiogram (MRA) with gadolinium contrast of the right lower limb revealed a false aneurysm of one of the terminal branches of the peroneal artery. In addition the peroneal artery was found to be anomalous, crossing the ankle joint and consisting of several large branches forming an anastomosis in the region (Figure 1).

**Figure 2.**
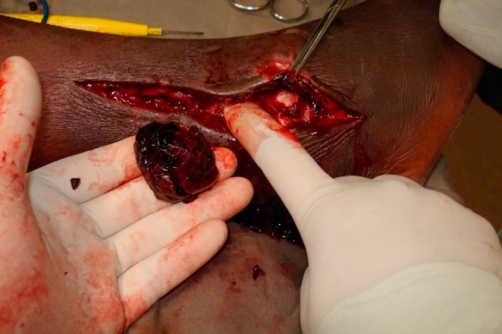
Exploration of the false aneurysm via longitudinal incision in the region of the lateral malleolus with digital pressure to control bleeding.

**Figure 3.**
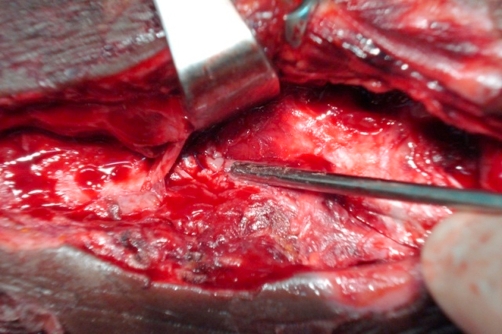
Distal feeding vessel oversewn with a 5.0 prolene suture.

Immediate exploration of the false aneurysm was undertaken via a longitudinal incision in the region of the lateral malleolus with digital pressure to control bleeding and the fine distal feeding vessel oversewn with a 5.0 prolene suture (Figure 2). Haemostasis was achieved and the wound closed (Figure 3). The necrotic skin of the region eventually sloughed and formed an ulcer, which slowly but fully healed over a 3-month period. The patient made an uneventful recovery.

## Discussion

True aneurysms of the peroneal artery are extremely rare with isolated reports of cases related to mycotic aneurysms [1] or connective tissue disorders such as Behcet’s disease [2]. Mycotic pseudoaneurysms typically involve major axial vessels proximal to the popliteal artery, however, there is an association with the tibioperoneal vessels in infective endocarditis, brucella canis [1] and gram-positive pathogens in intravenous drug Abusers [3]. A search of the literature reveals a handful of cases and one review of false aneurysms documented in various scenarios [15]. Trauma to the leg or ankle is the most common cause of such pseudoaneurysms specifically of the peroneal artery. It has been documented in ankle sprain/soft tissue injury, penetrating or blunt injury and malleolar fracture of the ankle [4-6]. Iatrogenic causes have also been described including thrombo-embolectomy using a fogarty balloon catheter [7] or after femoro-peroneal bypass grafting [8].

Management has traditionally been surgical, however, peroneal pseudoaneurysms may thrombose spontaneously [9,10]. Since the advent of modern techniques in interventional radiology it is now becoming more acceptable to perform coil embolization [11,12], thrombin injection [13] or stent insertion [14].

Surgical management involves the evacuation of the haematoma after proximal and distal vascular control has been achieved. The defect in the arterial wall can either be repaired by primary closure or by insertion of a vein patch. Vein interposition graft or prosthetic graft can also be used if the segment of the disrupted artery cannot be primarily repaired.

In this particular case there are two important learning points. Firstly, due to the location of this pseudoaneurysm just postero-inferior to the lateral malleolus it was technically challenging to get proximal control. Therefore, the procedure was done using pure digital control of the peroneal artery just proximal to the injured vessel branch. For extra safety a tourniquet was applied to the thigh, but not inflated as extra insurance in the event that digital control was insufficient.

The second point is noted on MRA in which there was an anatomical anomaly of the peroneal artery. The vessel appeared to cross the ankle joint and to have several large branches, which may have accounted for the injury to the vessel in this particular case.

It is recommended that an MRA or CTA be done prior to exploration for distal peroneal false aneurysms for two reasons; to define anatomy and plan surgical approach, since the vessel may not always be easily accessible and to document and possibly correlate anatomic anomalies of the vessel with this injury for future reference.

## Abbreviations

CTA: Computed Tomography Angiogram
MRA: Magnetic Resonance Angiogram
